# Diagnostic efficacy of apparent diffusion coefficient measurements in differentiation of malignant intra-axial brain tumors

**DOI:** 10.3906/sag-2006-1

**Published:** 2021-02-26

**Authors:** İlker EYÜBOĞLU, İmet Meriç ÇAKIR, Serdar ASLAN, Ahmet SARI

**Affiliations:** 1 Department of Radiology, Karadeniz Technical University, Faculty of Medicine, Trabzon Turkey; 2 Department of Radiology, Giresun University, Faculty of Medicine, Giresun Turkey

**Keywords:** Brain tumors, magnetic resonance imaging, diffusion-weighted imaging, apparent diffusion coefficient

## Abstract

**Background/aim:**

To evaluate diagnostic efficacy of the apparent diffusion coefficient measurements from tumor (ADCt) and tumor circumference hyperintensities (ADCtch) in different types of malignant intra-axial brain tumors.

**Materials and methods:**

Between April 2013 and June 2017 **,** 125 patients (52 females (41.6%) and 73 males (58.4%); mean age: 53 years, age range: 14-81 years), who underwent diffusion-weighted imaging (DWI) with intracranial mass, were retrospectively evaluated. The mean ADC_t_ and ADC_tch_ values and ratios were measured.

**Results:**

Of the 125 patients, 22 (17.6%) had a low-grade glioma (LGG), 55 (44%) had a high-grade glioma (HGG), 32 (25.6%) had metastasis, and 16 (12.8%) had lymphoma diagnosis. There was a statistically significant difference in LGG and HGG in terms of mean ADC_t_ and mean ADC_tch_ values, and ratios. ADC_tch_ values and ratios showed a statistically significant difference in the differentiation of HGG and metastasis and in the differentiation of HGG and lymphoma. According to ROC curve analysis, a cut-off value of 1.49 × 10−3 mm2/s for the mean ADC_tch_ value generated the best combination of 70% sensitivity and 71% specificity for differentiation of HGGs and metastasis. The mean ADC_tch_ value had the highest statistical predictive value for differentiation of HGGs and lymphoma with a sensitivity of 78% and a specificity of 76% for the optimal cut-off value of 0.82 × 10ˉ3 mm²/s.

**Conclusion:**

The mean ADC_t_ ratio allowed reliable differentiation of LGG and high grade brain tumors, including HGGs, metastases, and lymphoma. The mean ADC_tch_ might be a better imaging biomarker in the differentiation of HHG from metastasis and lymphoma.

## 1. Introduction

Magnetic resonance imaging (MRI) plays a critical role in the preoperative evaluation of brain tumors. However, since tumor groups generally show similar signal intensity and contrast enhancement patterns on brain MRI, advanced MRI studies are needed in terms of differentiation of tumor groups. Diffusion-weighted imaging (DWI) is used for the differentiation and grading of tumors on the basis of cellularity with the microscopic movement of water protons. The studies have reported an inverse correlation between cellularity and apparent diffusion coefficient (ADC) measurement [1].

It is a known fact that gliomas have regions of different histological grades within the tumor. Histologically, the region with the highest grade within the tumor (cellular atypia, vascularization, mitotic characteristic, and necrosis) indicates the true grade of the tumor. Therefore, knowing this region is of great importance in both predicting prognosis and planning the appropriate treatment [2].

As a result of local disruption of the blood-brain-barrier, vasogenic edema is present around most brain tumors. In addition to vasogenic edema, infiltrative cells are present in the peritumoral region of primary brain tumors. While high-grade gliomas (HGG) usually grow infiltratively and invade surrounding tissues, metastasis and lymphoma exhibit an expansive growth and cause displacement in tissues rather than invading surrounding brain tissues [1,3]. The aim of this study is to investigate the contribution of ADC values measured from the tumoral and peritumoral regions to differential diagnosis in primary and metastatic intra-axial brain tumors.

## 2. Materials and methods

This retrospective study was approved by local ethics committee (2015-50), and the requirement for patient informed consent was waived.

### 2.1. Study population

**Figure 1:**
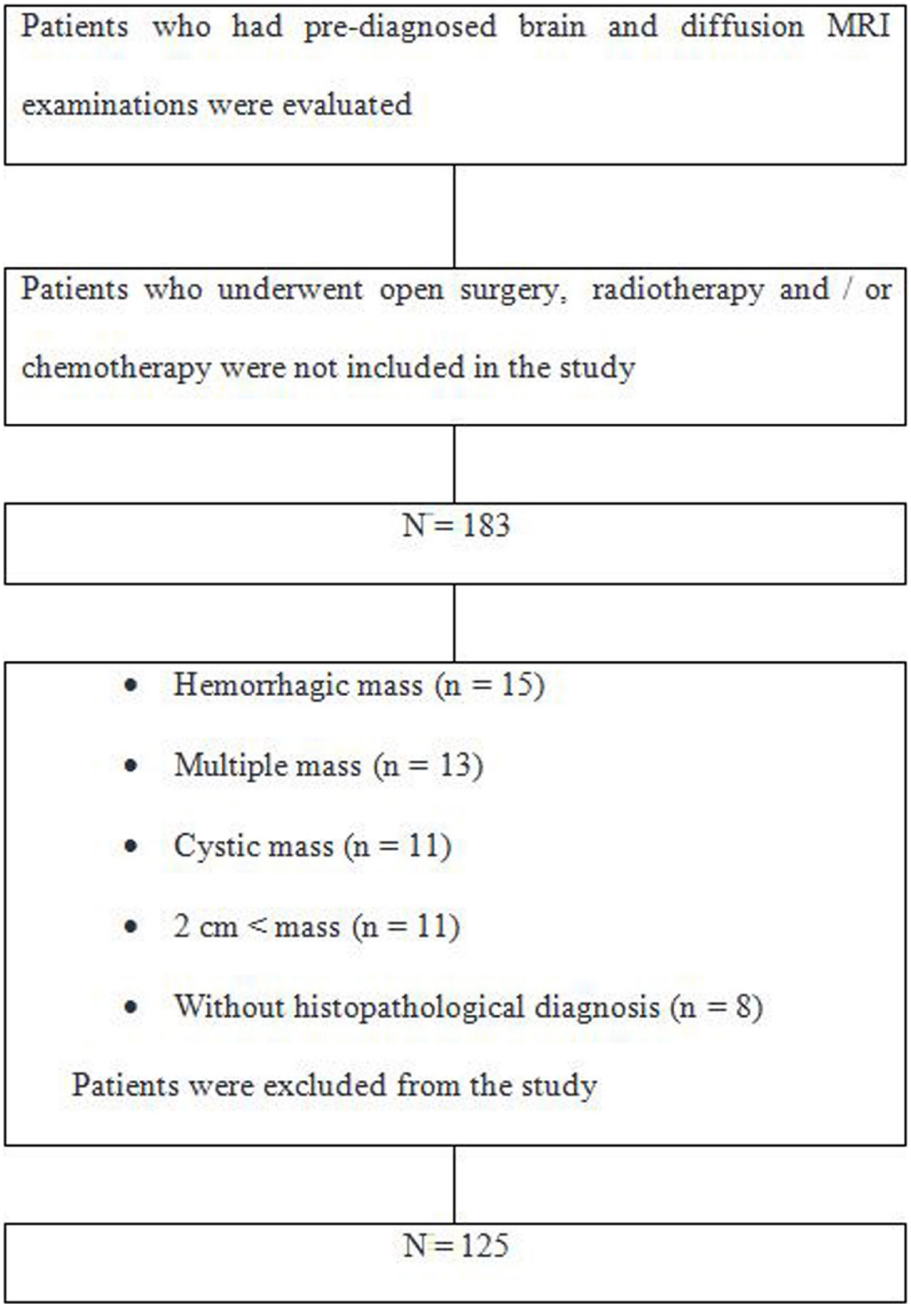
Flow diagram of the participants.

Between April 2013 and June 2017, MRI examinations of 183 patients, with a diagnosis of intracranial mass, were evaluated retrospectively. Patients who had undergone open surgery and stereotaxic biopsy before MRI and who received radiotherapy and/or chemotherapy were excluded from the study. Patients with hemorrhagic mass (n = 15), multiple masses (n = 13), cystic mass (n = 11), masses less than 2 cm for optimal DWI recon (n = 11) on MRI examination, and without pathological diagnosis (n = 8) were excluded from the study. The inclusion and exclusion criteria for the study are presented in the flow diagram (Figure 1).

### 2.2. Imaging protocol

All MRI examinations were performed with a 1.5 T MRI system (Symphony; Siemens Medical Systems, Erlangen, Germany) using a 4-channel head coil. Examinations were obtained using axial and sagittal T1-weighted spin-echo (SE) images (TR/TE, 495/9.6; NEX, 1; bandwidth, 300 Hz; matrix, 384 × 512; slice thickness, 5 mm; examination time, 3:30 min; and FOV, 280 × 83 mm), axial T2- weighted fast SE images (TR/TE, 4650/98; NEX, 1; bandwidth, 500 Hz; matrix, 384 × 512; slice thickness, 5 mm; examination time, 1:16 min; and FOV, 280 × 83 mm), and axial fluid-attenuated inversion recovery (FLAIR) sequence (TR/TE, 9580/125; matrix, 384 × 512; slice thickness, 5 mm; examination time, 4:11 min; the field of view (FOV), 280 × 83 mm). After the intravenous administration of 0.2 mg/kg gadolinium, contrast-enhanced T1A SE sequences were obtained in the axial, coronal, and sagittal planes.

DWI was obtained using a spin-echo sequence in the transverse plane with the single-shot echo-planar technique. The imaging parameters were as follows: TR/TE, 3200/94; bandwidth, 1345 Hz; matrix, 192 × 192; NEX, 3; slice thickness, 5 mm; interslice gap, 1.5 mm; examination time, 1:12 min; and FOV, 230 x 230 mm. The diffusion gradients were encoded in three orthogonal directions. Three different b values (0, 500 and 1000 s/mm²) were used for each slice. In all patients, ADC maps were obtained using the b values of 0 and 1000 s/mm².

### 2.3. Image analysis

**Figure 2:**
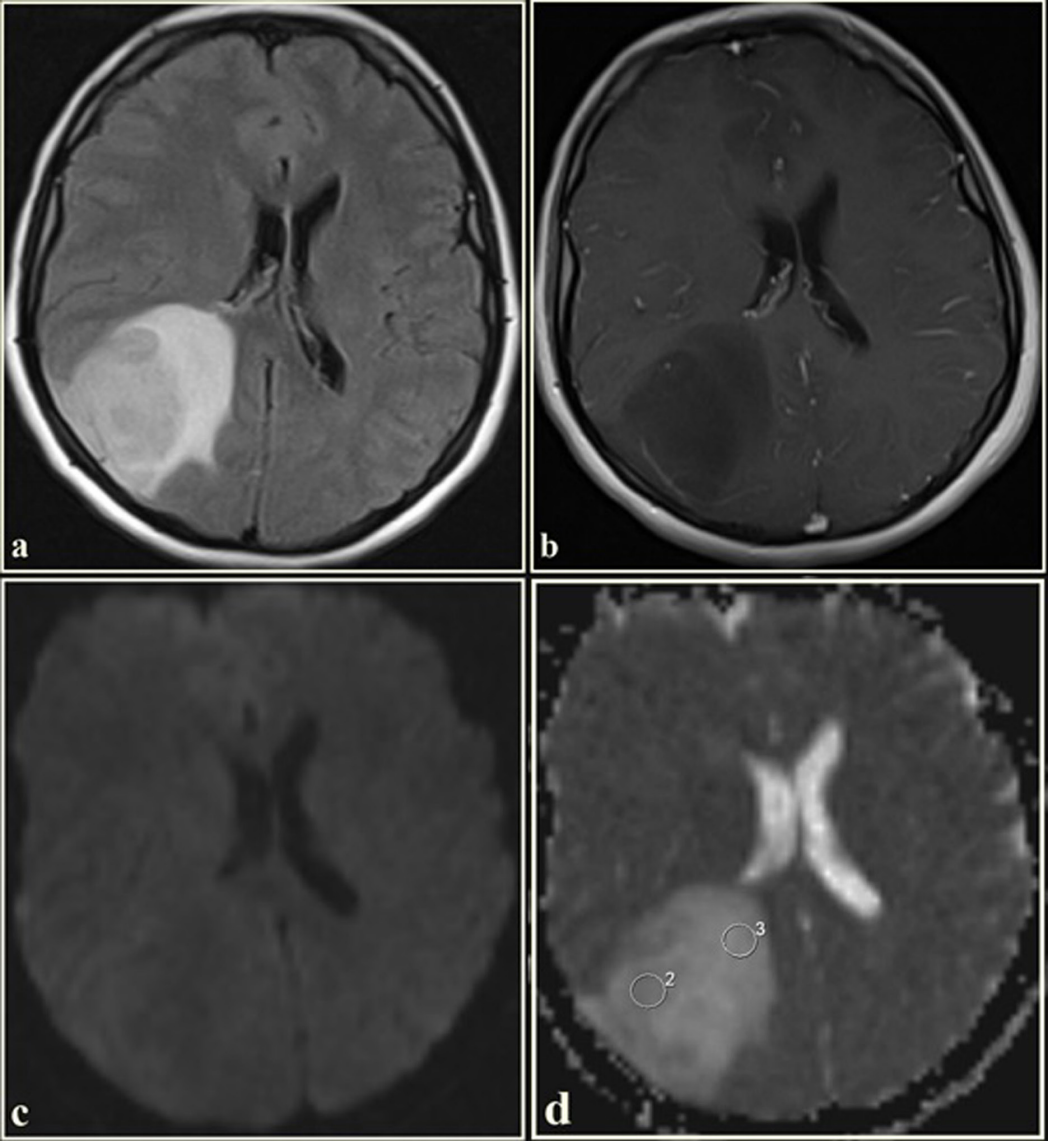
The FLAIR axial image of a 32-year-old female. (a) An isointense mass with tumor circumference hyperintensities in the right temporo-occipital lobe. The mass shows no contrast enhancement (b). On the diffusion-weighted imaging (c), the mass is intermediate signal intensity. The tumor and tumor circumference are hyperintense on the ADC map (d). The ADC value, measured from the tumoral region, was 1.51 × 10^−3^ mm^2^/s, and the ADC value, measured from the tumor circumference region, was 1.80 × 10^−3^ mm^2^/s. (Histopathological diagnosis: diffuse astrocytoma, WHO grade 2).

**Figure 3:**
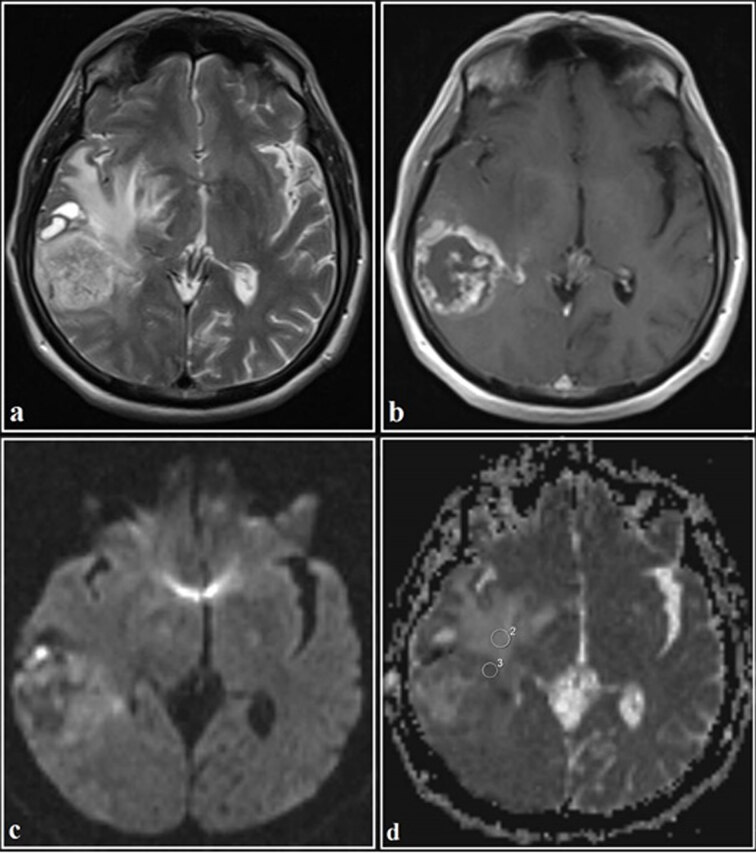
The axial T2WI of a 69-year-old male. (a) A heterogeneous hyperintense mass in the right temporal lobe. Tumor circumference is hyperintense. The lesion has a ring and nodular enhancement on the contrast-enhanced axial T1WI (b). On the diffusion-weighted imaging (c), while the tumor center is hypointense, its periphery is hyperintense. The tumor circumference is intermediate signal intensity. On the ADC map (d), the tumor circumference is heterogeneous hyperintense. The ADC value, measured from the tumoral regions, was 0.99 × 10^−3^ mm^2^/s, and the ADC value, measured from the tumor circumference hyperintensities, was 1.38 × 10^−3^ mm^2^/s (Histopathological diagnosis: glioblastoma multiforme, WHO grade 4).

**Figure 4:**
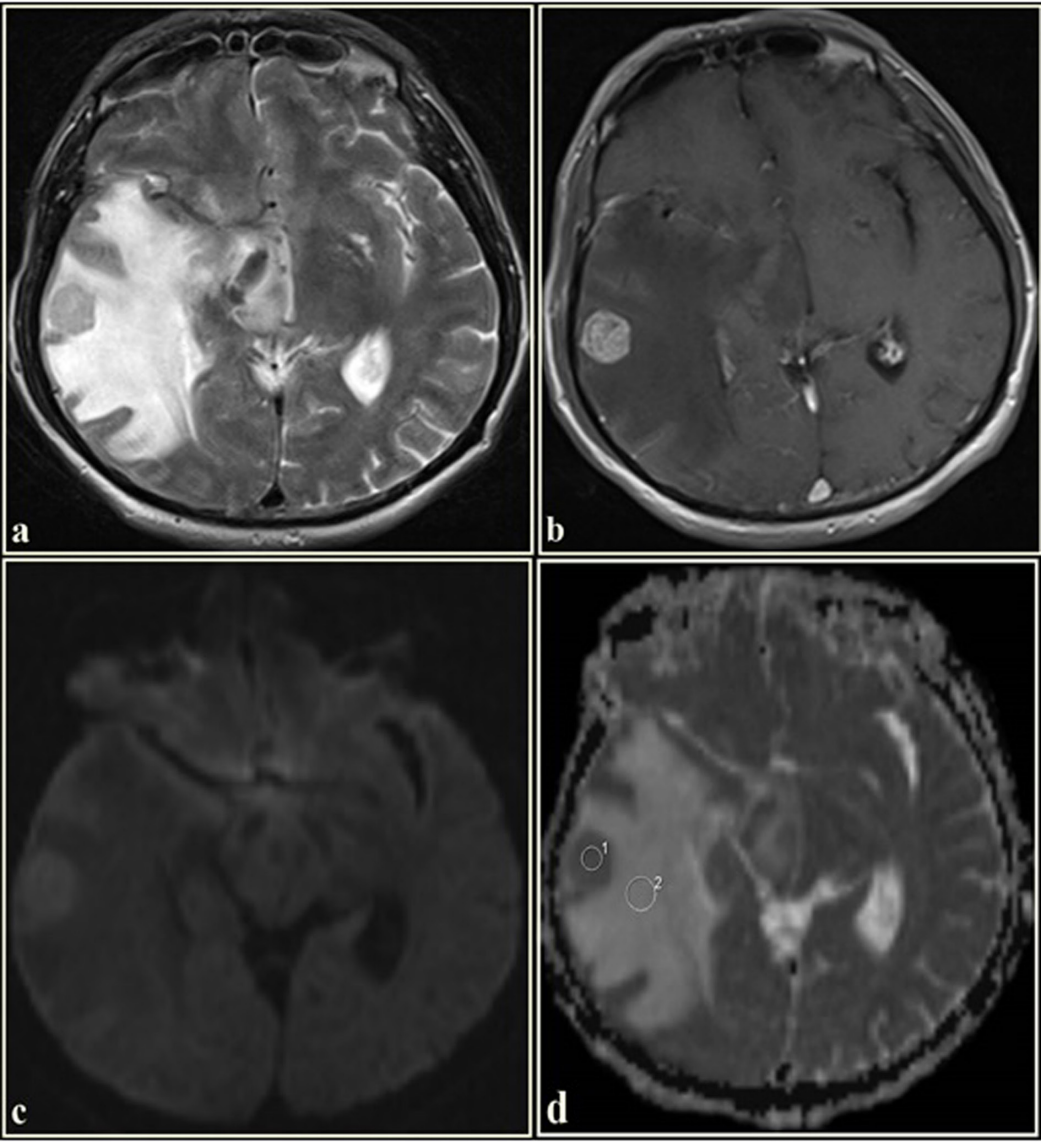
The axial T2WI of a 56-year-old male. (a). Shows a hyperintense mass in the right temporal lobe. Tumor circumference is hyperintense. The lesion is homogeneously enhancing on the postcontrast T1WI (b). On the diffusion-weighted imaging (c), while the tumor is hyperintense, its circumference is hypointense. The mass is hypointense on the ADC map (d). The tumor circumference is hyperintense. The ADC value, measured from the tumoral region, was 1.11 × 10^−3^ mm^2^/s, and the ADC value, measured from the tumor circumference hyperintensities, was 2.00 × 10^−3^ mm^2^/s (Histopathological diagnosis: lung adenocarcinoma metastasis).

**Figure 5:**
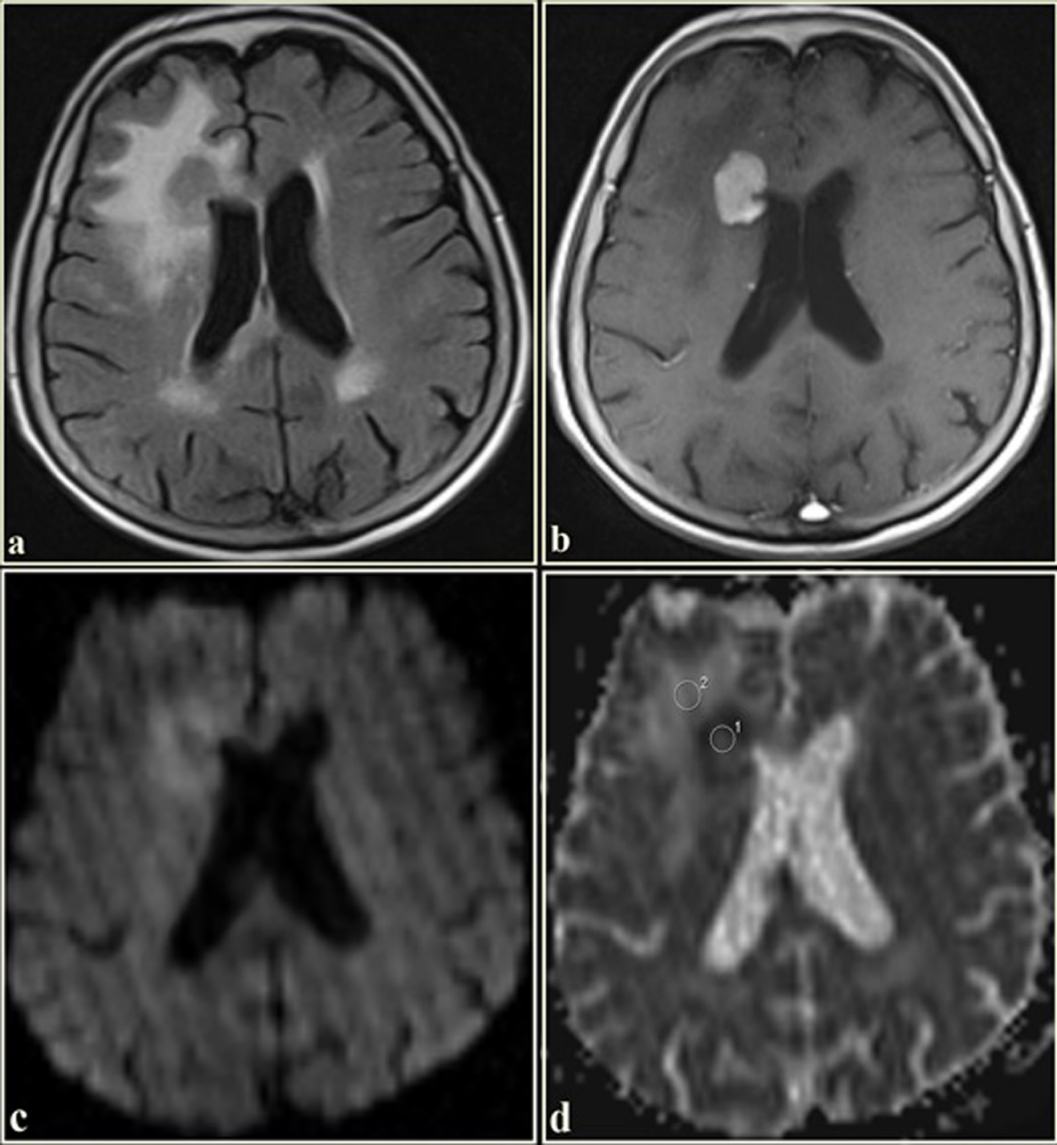
The axial T2WI of a 79-year-old female. (a) Shows an isointense mass in the right frontal lobe. The lesion is homogeneously enhancing on the post-contrast T1WI (b). On the diffusion-weighted imaging (c), the mass center shows low signal intensity. The tumor circumference shows relatively intermediate signal intensity. On the ADC map (d), the tumor is significantly hypointense while its circumference is hyperintense. The ADC value measured from the tumoral region was 0.48 × 10^−3^ mm^2^/s, and the ADC value, measured from the tumor circumference hyperintensities, was 1.37 × 10^−3^ mm^2^/s (Histopathological diagnosis: cerebral lymphoma).

ADC values were manually measured by 2 experienced radiologists (İ.E. and İ.M.Ç. with 9 and 4 years of experience, respectively) on the workstation (Leonardo, Siemens Healthcare) independently of each other, who were unaware of the clinical knowledge of the patients and the pathological diagnosis of the masses. We selected all continuous sections that included enhancing tumor and the peritumoral region. The lowest ADC areas were determined by visual inspection. One large and 2 small, uniform, and round (50–100 mm2), ROIs were drawn manually to the lowest ADC areas. The ROI with the lowest ADC was chosen from these ROIs as the minimum ADC (ADCmin). The ROI with the highest ADC was chosen from these ROIs as the maximum ADC (ADCmax). The average of ROIs was recorded as mean ADC (ADCmean). DWI was used only in qualitative analysis with conventional MR sequences. ROIs were carefully placed in the most homogeneous solid tumor area (ADCt) corresponding to the enhancing area, nonenhancing tumor circumference hyperintensities (ADCtch), and contralateral normal white matter (ADCn) on the ADC map. In nonenhancing tumors, ADC_t_ value was measured by taking the most homogeneous parts of the tumor into account on the T1 and T2-weighted image. In all regions, mean ADC values (ADCt, ADCtch, and ADCn) were recorded. The cystic, necrotic, or hemorrhagic parts of the lesions were not included in the circular ROI images (Figures 2–5). When there was discordance in ADC values between radiologists, consensus was reached by a collaborative decision.

ADC ratios were calculated by dividing ADC values of tumoral and peritumoral regions by ADC value of contralateral normal white matter (ADCt/n and ADCtch/n). 

### 2.4. Statistical analysis

Statistical analyses were performed using the SPSS software, version 21 (IBM Corporation, Armonk, NY, USA). Interobserver agreement values were determined using Pearson correlation test with a 95% confidence interval: 0–0.20 inadequate agreement; 0.21–0.4 slight agreement; 0.41–0.6 moderate agreement; 0.61–0.8 substantial agreement, 0.81–1.00 almost perfect agreement, and 1.00 perfect agreements. ANOVA calculation was used to determine the statistically significant difference of the four tumor groups (LGG, HGG, and metastasis and primary cerebral lymphoma) according to their different ADC values and ratios. Significance values were tested based on a 95% confidence interval for the difference between groups. If the P value was < 0.05, differences were considered significant. The multiple comparisons Tukey’s T-procedure was used for further examination of pairwise differences of significant ADC values. If the P value was < 0.05, differences were considered significant.

In the differentiation of the tumor groups, receiver operating characteristic (ROC) analysis was used to determine the threshold value of ADC with the best sensitivity and specificity combination.

## 3. Results

The study group consisted of a total of 125 patients (52 females and 73 males) with a mean age of 53 (14–81 years) years, which had solitary intra-axial brain tumor.

In the histopathological evaluation, 22 (17.6%) patients were diagnosed with LGG (grade 2 oligodendroglioma = 6, grade 2 astrocytoma = 14, ependymoma = 2), 55 (44%) patients with HGG (anaplastic oligodendroglioma = 3, anaplastic astrocytoma = 3, glioblastoma multiforme = 49), and 16 (12.8%) patients were diagnosed with primary cerebral lymphoma. 32 (25.6%) patients were diagnosed with metastasis (lung cancer = 18, breast cancer = 5, malignant melanoma = 2, seminoma= 1, nasopharyngeal cancer = 1, bladder cancer = 1, cervical cancer = 1, endometrial cancer = 1, thyroid anaplastic cancer = 1, colon cancer = 1) (Table 1). Histopathological diagnosis was present in all primary intracranial masses, lymphomas, and metastasis cases. 

**Table 1:** Pathological diagnoses of brain tumors.

Tumors	n (%)	Subgroups (n)	
LGG	22 (17.6%)	Oligodendroglioma (n = 6)	
		Astrocytoma (n = 14)	
		Ependymoma (n = 2)	
HGG	55 (44%)	Anaplastic oligodendroglioma (n = 3)	
		Anaplastic astrocytoma (n = 3)	
		Glioblastoma multiforme (n = 49)	
Lymphoma	16 (12.8%)	Diffuse large B-cell lymphomas (n = 16)	
Metastases	32 (25.6%)	Lung cancer (n = 18)	Breast cancer (n = 5)
		Malignant melanoma (n = 2)	Seminoma (n = 1)
		Nasopharyngeal cancer (n = 1)	Bladder cancer (n = 1)
		Cervical cancer (n = 1)	Endometrial cancer (n = 1)
		Thyroid cancer (n = 1)	Colon cancer (n = 1)

n = number of cases and in between parenthesis given the percentages of cases.

Interobserver agreement values of ADC_t_ and ADCn were almost perfect (0.82, P < 0.001; and 0.85, P < 0.001, respectively). Interobserver agreement value of ADC_tch_ was moderate (0.58, P = 0.024).

There was a statistically significant difference in all parameters (ADC values and ratios) in 4 tumor groups with the ANOVA test (P < 0.05) (Table 2). ADC_t_ values and ratios were higher in the LGG than in the other tumor groups. ADC_tch_ values and ratios were lower in the HGG tumors than in the other tumor groups. The lowest ADC_t_ values were measured in lymphoma.

**Table 2:** Mean ADC values of tumor and tumor circumference hyperintensities in different tumor types, ratio of ADC value of tumoral, and tumor circumference hyperintensities to contralateral normal white matter ADC value.

	LGG	HGG	Metastasis	Lymphoma	P value
ADC_t_ mean	1.238 ± 0.357	0.965 ± 0.238	1.025 ± 0.468	0.744 ± 0.102	< 0.001
ADC_t_ ratio mean	1.726 ± 0.521	1.283 ± 0.351	1.334 ± 0.537	0.972 ± 0.150	< 0.001
ADC_tch_ mean	1.572 ± 0.242	1.373 ± 0.253	1.605 ± 0.186	1.670 ± 0.160	< 0.001
ADC_tch_ ratio mean	2.195 ± 0.346	1.822 ± 0.375	2.128 ± 0.326	2.184 ± 0.287	< 0.001

Note: t = tumor; tch = tumor circumference hyperintensities; ADC_t_ ratio = tumor/contralateral hemisphere. ADCtch

In the pairwise discrimination tests conducted with the Tukey’s T-procedure, there was a statistically significant difference LGG and HGG in terms of mean ADC_t_ and mean ADC_tch_ values and ratios. Mean ADC_t_ ratios showed a statistically significant difference in differentiation of LGG and metastasis. ADC_t_ values and ratios showed a statistically significant difference in the differentiation of LGG and lymphoma and in the differentiation of metastasis and lymphoma. Finally, ADC_tch_ values and ratios showed a statistically significant difference in the differentiation of HGG and metastasis and in the differentiation of HGG and lymphoma (P < 0.05) (Table 3).

**Table 3:** Multiple comparisons with Tukey t-Procedure tumor and tumor circumference hyperintensities ADC values and ratios in different tumor types.

Comparison	Mean ADCt	Mean ADC_t_ ratio	Mean ADCtch	Mean ADC_tch_ ratio
LGG and HGG	0.006	0.000	0.004	0.000
LGG and metastasis	0.086	0.006	0.952	0.898
LGG and lymphoma	0.000	0.000	0.541	0.999
HGG and metastasis	0.842	0.948	0.000	0.001
HGG and lymphoma	0.071	0.044	0.000	0.002
Metastasis and lymphoma	0.023	0.026	0.777	0.949

Note: LGG = low grade glioma; HGG = high grade glioma; t = tumor; tch = tumor circumference hyperintensities. ADC_t_ ratio = tumor/contralateral hemisphere; ADCtch

**Figure 6:**
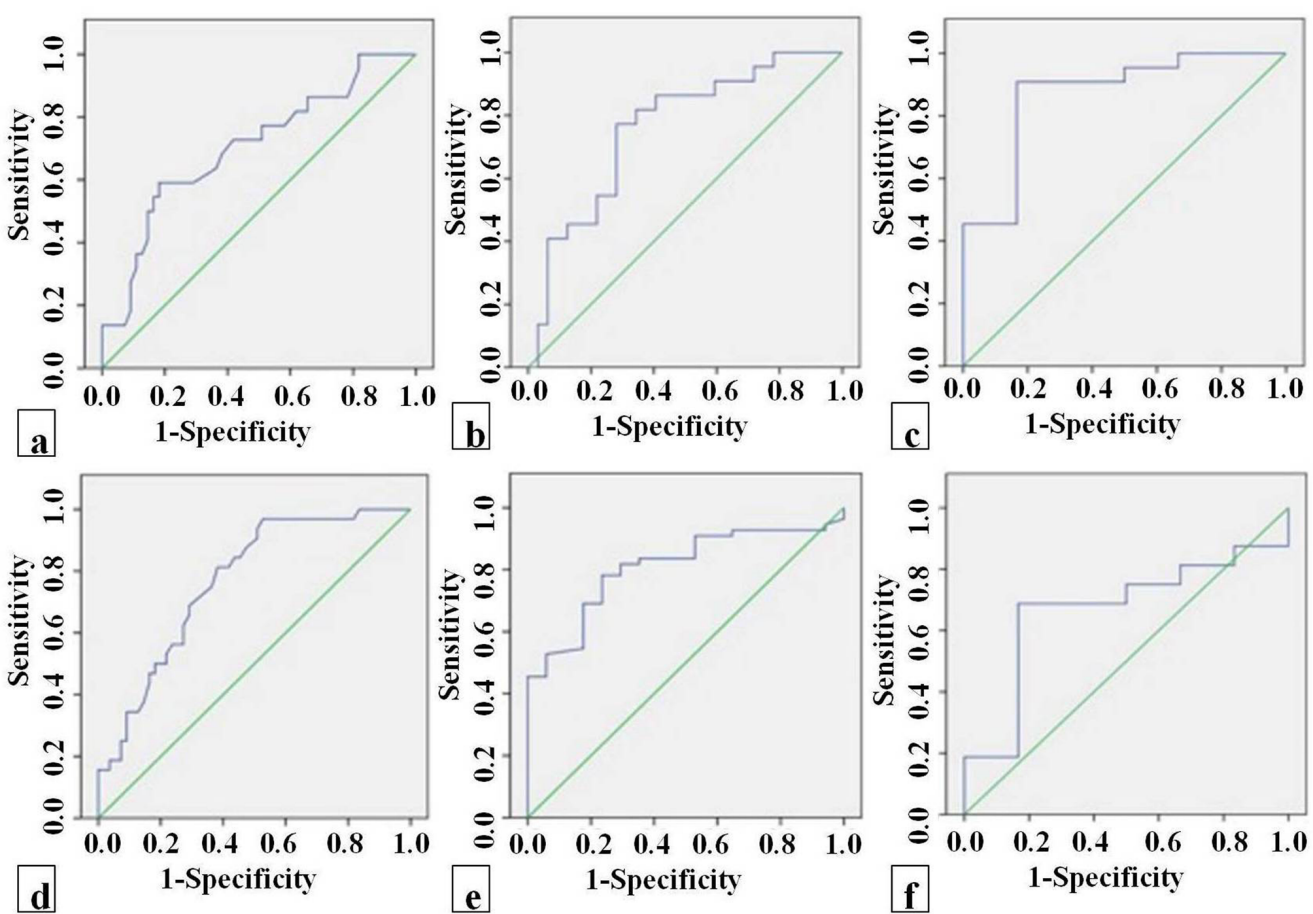
(a). The most powerful predictive marker to differentiate between LGG and HGG was the ADC_t_ ratio; the area under the ROC curve (AUC) was 0.778 (670-899 with a 95% confidence interval) (b). The most powerful predictive marker to differentiation of LGG and metastasis was the mean ADC_t_ ratio; the AUC was 0.760 (630-890 with a 95% confidence interval) (c). Th e most powerful predictive marker to differentiation of LGG and lymphoma was the mean ADC_t_ ratio; the AUC was 0.968 (919-1017 with a 95% confidence interval) (d). The most powerful predictive marker to differentiation of HGG and metastasis was the mean ADC_tch_ value; the AUC was 0.7767 (681-887 with a 95% confidence interval) (e). The most powerful predictive marker to differentiation of HGG and lymphoma was the mean ADC_tch_ value; the AUC was 0.808 (704-912 with a 95% confidence interval) (f). The most powerful predictive marker to differentiation of metastasis and lymphoma was the mean ADC_t_ value; the AUC was 0.778 (654-914 with a 95% confidence interval).

In the pairwise discrimination tests, a ROC analysis was performed for each single factor ADC value found statistically significant between both tumor groups. The most powerful predictive marker to differentiation of LGG and HGG was the mean ADC_t_ ratio, with a sensitivity of 72% and a specificity of 72% for the optimal cut off value 2.06 × 10ˉ³ mm²/s. The most powerful predictive marker to differentiation of LGG and metastasis was the mean ADC_t_ ratio, with a sensitivity of 77% and a specificity of 72% for the cut off value 1.44 × 10−3 mm²/s. The most powerful predictive marker to differentiation of LGG and lymphoma was the mean ADC_t_ ratio, with a sensitivity of 90% and a specificity of 94% for the cut off value 1.21 × 10−3 mm²/s. The most powerful predictive marker to differentiation of HGG and metastasis was the mean ADC_tch_ value, with a sensitivity of 70% and a specificity of 71% for the cut off value 1.49 × 10−3 mm²/s. The most powerful predictive marker to differentiation of HGG and lymphoma was the mean ADC_tch_ value, with a sensitivity of 78% and a specificity of 76% for the cut off value 0.82 × 10−3 mm²/s. Finally, the most powerful predictive marker to differentiation of metastasis and lymphoma was the mean ADC_t_ value, with a sensitivity of 72% and a specificity of 82% for the cut off value 1.13 × 10−3 mm²/s (Figure 6). 

## 4. Discussion

In our study, we evaluated diagnostic performance with ADC measurements to facilitate the preoperative differentiation of malignant intra-axial brain tumors, which are usually not distinguishable with conventional MR imaging sequences. ADC_t_ values and ratios were higher in LGGs than in other tumor groups. Our results showed that the most important parameter is the mean ADC_t_ ratio, distinguishing LGGs from high grade brain tumors, including HGGs, metastases, and lymphoma. The lowest ADC_tch_ values and ratios were measured in HGGs. According to the ROC curve analysis, a cut-off value of 1.49 × 10−3 mm2/s for the mean ADC_tch_ value generated the best combination of sensitivity 70% and specificity 71% (P < 0.05) for differentiation of HGGs and metastasis. The mean ADC_tch_ value had the highest statistical predictive value for differentiation of HGGs and lymphoma with a sensitivity of 78% and a specificity of 76% for the optimal cut-off value of 0.82 × 10−3 mm²/s. The most powerful predictive value, for differentiation of metastasis and lymphoma, was the mean ADC_t_ value, with a sensitivity of 72% and a specificity of 82% for the optimal cut-off value of 1.13 × 10−3 mm²/s. 

DWI has been used to grade or differentiate among brain tumors on the basis of cellularity. The ADC measurements reflect the mobility of the free water fraction, including extracellular and intracellular water, within the tissue. Many studies have shown that calculation of ADC may help in differentiating cerebral tumors [1,4]. In addition, an inverse correlation has been found between tumor cellularity and ADC values [5,6].

Most brain tumors are surrounded by vasogenic edema that showing high signal intensity on T2WI. Vasogenic edema is the most common form of cerebral edema in brain tumors. There is a tendency for local disruption in the blood-brain barrier. Due to the increased capillary permeability and pressure, plasma fluid and protein accumulate from the vascular space to the extracellular space. Tumor circumference is pure vasogenic edema in metastatic brain tumors or lymphoma, and there are no tumor cells in tumor circumference. On the other hand, the tumor circumference hyperintensities are infiltrative edema in high-grade glioma. There are infiltrative tumoral cells that infiltrate through the blood brain-barrier and may invade white matter pathways [2].

Several previous studies [7,8] reported that ADC values were not useful in preoperative grading of glioma. In our study, the mean ADC_t_ ratio had the highest statistical predictive value in the differentiation of LGGs and HGGs, with a sensitivity of 72% and a specificity of 72% for the optimal cut off value of 2.06 × 10−3 mm²/s. A recent study on the mean ADC of brain tumors showed that the mean ADC_t_ values are significantly higher in LGG than in HGG. The cut off value of 1.40 × 10−3 mm²/s can be considered an index for ADC to distinguish HGG from LGG. In addition, they emphasized that standard deviation of ADC is also substantial in differentiation of LGG and HGG [9]. Cihangiroglu MM et al. [10] compared DWI using high b-value (b = 3000 s/mm2) to standard b-value (b = 1000 s/mm2) in the preoperative grading of supratentorial gliomas. They found that ADC parameters derived from DW-MRI using a high b-value allow a better differential diagnosis of gliomas, especially for differentiating grades III and IV, than those derived from DW-MRI using a standard b-value. A possible reason for this result is that the tumor boundaries were directly determined by the DWI maps with b = 3000 s/mm2 [11].

In our study, the mean ADC_t_ ratio had the highest statistical predictive value in the differentiation LGG and metastasis, with a sensitivity of 77% and a specificity of 72% for the cut off value of 1.44 × 10−3 mm²/s. The mean ADC_t_ ratio had the highest statistical predictive value in the differentiation LGG and lymphoma, with a sensitivity of 90% and a specificity of 94% for the cut off value of 1.21 × 10ˉ³ mm²/s. These results show us that tumoral cellularity is higher in metastasis and lymphoma than in LGG. 

There was no statistically significant difference between LGGs and metastasis, between LGG and lymphoma, and between metastasis and lymphoma in terms of peritumoral ADC values and ratios (P > 0.05). These values show us that there is pure vasogenic edema in the tumor circumference hyperintensities of LGGs, metastasis, and lymphoma.

The previous studies reported that ADC_t_ values are not useful in differentiation of HGG and metastasis [12–14]. However, Krabbe et al. and Lee EJ et al. reported that ADC_t_ value was higher in cerebral metastasis than in HGGs [6,15]. They stated that the high value in metastases was due to the high content of intracellular or extracellular water in metastases compared to gliomas. In our study, there was no statistically significant difference between HGG and metastasis in terms of ADC_t_ values and ratios.

HGG typically shows an infiltrative growth pattern with invasion of the surrounding brain tissues. However, brain metastasis reveals an expansive growth pattern and displaces the surrounding brain tissues [15]. Some researchers hypothesized that ADC_tch_ values could be used to distinguish infiltrative edema of the glioma from metastatic vasogenic edema [1,15]. But, some conflicting results were reported in these studies. Pierre Lemercier et al. used the gradient of ADC values in the tumor circumference hyperintensities to differentiate HGG from metastases. ADC measurements were made in areas near, an intermediate distance from, and far from the core-enhancing tumor (G1, G2, and G3). They found a gradient of ADC in the peritumoral edema of glioblastoma associated with peritumoral glial alterations [3]. While, Eun Ja Lee et al. reported that the mean minimum ADC values and mean ADC ratios in the tumor circumference of glioblastomas were significantly higher than those in metastases [15], Ionut Caravan et al. reported that the mean ADCmin values in the tumoral circumference of HGGs were significantly lower than those in brain metastases [16]. These conflicting results revealed that assessment of the tumor circumference hyperintensities with DWI could be challenging. Because, some high-grade tumor, such as primary (de-novo) glioblastoma, show minimal microscopic tumor infiltration in the tumor circumference hyperintensities. A meta-analysis study reported that DWI showed a moderate diagnostic performance for differentiation of HGG from brain metastasis [17]. In our study, ADC_tch_ values and ratios were lower in HGG than metastases, and there was a statistically significant difference (P < 0.05).

Authors of several studies [9, 18–20] have reported that DWI allows differentiation of lymphoma and HGG on the basis of ADC measurements. ADC_t_ and ADC_tch_ values and ratios for patients with lymphoma were substantially lower than those for patients with HGG. Although lymphomas had lower ADC_t_ values compared to HGGs in our study, there was no statistically significant difference (P > 0.05). However, a cut-off value of 0.82 × 10−3 mm2/s for the mean ADC_tch_ value generated the best combination of sensitivity 78% and specificity 76% (P < 0.05) for differentiation of HGGs and lymphoma. According to several authors, the differences in ADC between patients with lymphoma and those with metastasis were not statistically significant, although the ADC for patients with lymphoma was lower than that for patients with metastasis [2,14]. The lower ADC_t_ values in patients with lymphoma may have been related to the high degree of cellularity of these tumors leading to more restricted diffusion compared with that of patients with other malignant brain tumors [19–21]. In our study, the most powerful predictive marker was the mean ADC_t_ value in differentiation of metastasis and lymphoma.

In a variety of advanced noninvasive MRI techniques such as spectroscopy, perfusion imaging, and diffusion tensor imaging, we only evaluated the usefulness of ADC measurements in the differentiation of intra-axial brain tumors because DWI is the cost-effective and least time-consuming technique available in most hospitals. Data processing is fast and relatively easy [15].

Our study has several limitations. Firstly, the accuracy of statistical data varies due to the numerical inhomogeneity of the current patient groups. Secondly, the ROI placement method is subjective and may differ among researchers. Thirdly, the geometric resolution of the ADC maps is lower than conventional MRI. Therefore, some difficulties are encountered in ROI placement, especially in small-sized tumors or ring-enhancing tumors.

In conclusion, DWI with ADC measurements contribute significantly to conventional MRI in grading glial tumors and differentiating HGG from metastasis and lymphoma. Detecting peritumoral invasion and grading glial tumors, in the preoperative period, prevent unnecessary stereotaxic biopsy, help the surgeon in preoperative planning and may contribute to decrease in mortality and morbidity.
